# Preferred Orientation of Hydroxyapatite Ceramics Along the *c*-Axis Promotes Osteoblast Differentiation

**DOI:** 10.3390/ijms252312926

**Published:** 2024-12-01

**Authors:** Kitaru Suzuki, Masaki Tamazawa, Erika Onuma, Michiyo Honda, Mamoru Aizawa

**Affiliations:** 1 Department of Applied Chemistry, School of Science and Technology, Meiji University, Kawasaki 214-8571, Kanagawa, Japan; masakit21@outlook.jp (M.T.); e.oonuma@aist.go.jp (E.O.); michiyoh@meiji.ac.jp (M.H.); 2 Meiji University International Institute for Materials with Life Functions, Meiji University, Kawasaki 214-8571, Kanagawa, Japan

**Keywords:** hydroxyapatite, anisotropy control, crystal plane, osteoblast, bone differentiation

## Abstract

Hydroxyapatite (HAp) is similar to the main inorganic components of bone and tooth enamel. Furthermore, it possesses biocompatibility, making it suitable for clinical use in artificial bones. This study aimed to verify whether the preferred orientation of HAp influences osteogenesis. Using the templated grain growth method, we successfully fabricated HAp ceramics with a preferred orientation to *m* (*a*)-planes (aHAp) and examined the effects of this orientation on bone differentiation. Osteosarcoma-derived osteoblasts (MG-63) were cultured on aHAp and HAp ceramics made from commercially available powder (iHAp). Electron backscatter diffraction analysis revealed the crystal orientation distribution of HAp ceramics and the numerous exposed *a*-planes of aHAp. The MG-63 cultured on aHAp exhibited significantly higher alkaline phosphatase activity, a marker of early bone differentiation, compared to iHAp. Furthermore, the two-dimensional electrophoresis results indicated that the expressed proteins differed between aHAp and iHAp. These results indicate that controlling HAp’s crystal structure may promote the osteogenic potential of osteoblasts. In this study, we propose that the *a*-plane of HAp promotes bone differentiation during the early stages, presenting a promising approach for novel biomaterials, such as high-performance artificial bones.

## 1. Introduction

Hydroxyapatite (Ca_10_(PO_4_)_6_(OH)_2_: HAp) is known for its similarity to the main inorganic components of bone and tooth enamel [1,2]. In addition, HAp has anisotropy, with different properties that depend on the exposed crystal planes. HAp has a hexagonal crystal structure. It is also known to be positively charged when the *m* (*a*)-plane (in accordance with convention, hereafter, it will be written as *a*-plane) is exposed and negatively charged when the *c*-plane is exposed. In general, a long tubular bone has several exposed *a*-planes of HAp, whereas tooth enamel has several exposed *c*-planes of HAp [3]. HAp has also been used as a biomaterial, particularly as an artificial bone [4] and scaffold for hard-tissue regeneration [5,6], because of its biocompatibility.

In improving the bone-formation ability of existing HAp scaffolds for bone tissue regeneration, many efforts have been made to introduce ions and promote bone formation because the crystal structure of HAp is easily substituted with various ions. For example, strontium ions can improve bone formation by suppressing osteoclast activity, and silicate ions can enhance osteoblast activity [7,8]. Carbonate apatite has also been studied in recent years, as the minerals of a biological bone contain carbonate ions [9]. According to some reports, biomaterials with antibacterial properties can be developed by applying the aforementioned methods and substituting silver ions and copper ions [10]. A previous study has also reported that the loading of proteins, such as bone morphogenetic protein-2, can enhance the bone-formation ability [11,12]. However, controversy exists regarding its application because of the side effects observed in the spinal cord [12,13]. Furthermore, the use of local high-dose BMPs may cause various complications, such as ectopic bone formation [14].

In this study, we aimed to determine whether the preferred orientation of HAp affects bone differentiation in vivo. We have synthesized particles with each crystalline plane (*a*-plane or *c*-plane) of HAp exposed. In particular, apatite particles with many exposed *a*-planes (apatite fibers, hereafter, AF) have been used as a three-dimensional (3D) scaffold for tissue regeneration [15,16]. We have also synthesized HAp particles with many exposed *c*-planes [17] and reported that such particles with several exposed *a*-planes and *c*-planes adsorb different proteins [18]. As for the control of HAp crystal morphology, the fibrous HAp particles with preferred orientation to the *a*-plane have been synthesized by the hydrothermal method, hydrothermal homogeneous precipitation method, electrochemical method, and other methods until now [19,20,21]. Attempts to coat HAp layers with preferred orientation to the *c*-plane on metal have also been reported [22]. In addition to our own work, there have been reports of attempts to synthesize plate-like HAp particles with preferred orientation to the *c*-plane via electrochemical methods; however, these are very few reports [23]. Based on previous reports, a surface-polarized apatite is also one approach that can be used to modify surface properties [24]. Previously, we had successfully fabricated HAp ceramics with a preferred orientation to the *a*-plane (aHAp) using the templated grain growth (TGG) method, which could fabricate dense ceramics and maintain the orientation of the starting material [17,25,26]. We found that the surface potential and other properties of aHAp differ from those of ceramics made from commercially available HAp powder (iHAp) and that aHAp and iHAp ceramics can absorb different proteins. Furthermore, our findings indicate that proteins adsorbed onto aHAp have a beneficial effect on bone formation [27]. However, the effect of the crystal plane of HAp on bone formation remains unclear.

In this study, we clarified how the *a*-plane of HAp influences the level of bone differentiation in osteoblasts by culturing osteosarcoma-derived osteoblasts (MG-63) on aHAp that mimics the structure of a long tubular bone. The initial cell adhesion, cell proliferation, and osteo-differentiation potential of the osteoblasts to aHAp and iHAp were also examined. In addition, proteins were separated using two-dimensional (2D) electrophoresis to identify potential differences in the proteins expressed by the cells cultured on each specimen.

## 2. Results

### 2.1. Material Properties of the Starting Powder and Resulting Ceramics

**Figure 1:**
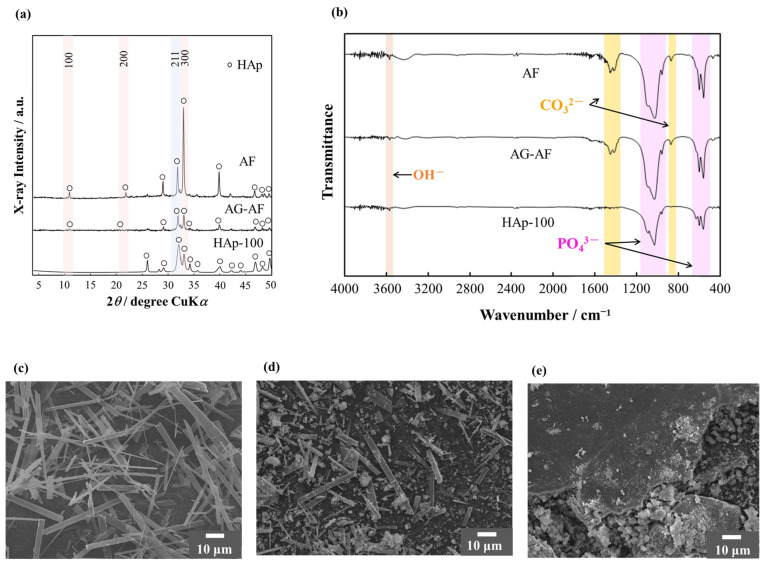
Characterization of AF, AG-AF, and HAp-100 powder: (**a**) X-ray diffraction (XRD) patterns and (**b**) FTIR spectra. Particle morphology of (**c**) AF, (**d**) AG-AF, and (**e**) HAp-100 powder.

The X-ray diffraction (XRD) patterns of the starting powders are shown in Figure 1a. Figure 1b–e show the starting powders obtained by Fourier transform infrared spectrometry (FTIR) and scanning electron microscopy (SEM), respectively. Figure 1a indicates that all of the starting powders treated in this study were single-phase HAp. The HAp-100 powder had typical diffraction peaks of HAp (ICDD-PDF Card No. 00-009-0432), and the peak corresponding to (300) of AF was higher than that of the HAp-100 powder showing preferential orientation of the *a*-plane. However, in the case of apatite gels and AF (AG-AF), the intensity slightly decreased. The FTIR results of the starting powders were assigned to the typical functional groups of HAp, such as the OH group and PO_4_ group, in addition to the CO_3_ group (Figure 1b). Figure 1c–e show the SEM images of the particle morphology of AF, AG-AF, and HAp-100 powder. The AF had a crystal structure with a fibrous morphology (Figure 1c). As shown in Figure 1d, the AG-AF had a similar structure to precipitated HAp microcrystals with a size of ~1 μm. The HAp-100 powder was composed of particles with various sizes, ranging from 1 to 10 μm and ≥50 μm (Figure 1e).

**Figure 2:**
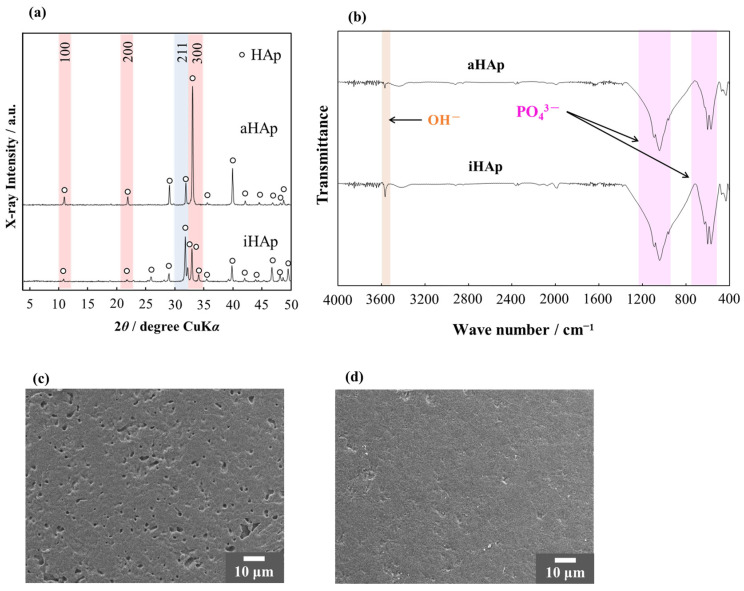
Characterization of aHAp and iHAp ceramics: (**a**) XRD patterns and (**b**) FTIR spectra. SEM images of (**c**) aHAp and (**d**) iHAp.

Figure 2 shows the characterization results of aHAp and iHAp ceramics. As shown in Figure 2a, both ceramics remained single-phase HAp. The diffraction of the plane corresponding to (300) of HAp in aHAp was stronger than that in iHAp. As shown in Figure 2b, both ceramics contained typical functional groups of HAp. Figure 2c,d show the SEM images of the surface of aHAp and iHAp. Few pores were found on any of the ceramic surfaces, and sintering had progressed. The relative density of aHAp was 90.63% (*n* = 6, with a standard deviation (SD) of 0.9409), and the relative density of iHAp was 95.24% (*n* = 7, SD = 0.3107).

**Figure 3:**
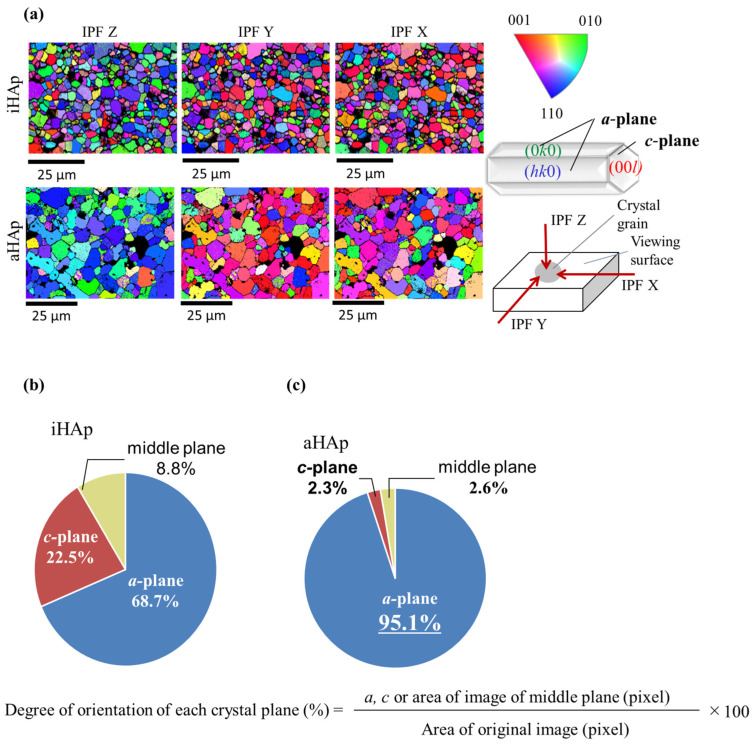
Crystal orientation distribution and quantitative evaluation of the degree of orientation for the fabricated ceramics: (**a**) EBSD images of the ceramics (The *a*-plane of HAp indicates green or blue, and the *c*-plane indicates red in this study) and (**b**) quantitative results of the crystal orientation distribution map of iHAp and (**c**) aHAp (the degree of orientation of each crystal plane was calculated using the above formula).

As shown in Figure 3a, the results of electron backscatter diffraction (EBSD) analysis indicated the distribution of the crystal orientation of HAp ceramics. The *a*-plane of HAp indicates green or blue, and the *c*-plane indicates red. When the images of the *Z*-axis (IPF Z) were focused, the iHAp crystal grains on the ceramics surface were shown in red, green, and blue. On the contrary, almost all the crystal grains of aHAp were shown in green and blue. Based on the results of the quantitative evaluation to calculate the ratio of the *a*-plane in ceramics using image analysis software, aHAp (95.1%) had higher values than iHAp (68.7%; Figure 3b,c).

### 2.2. Dissolution Tests of Ca^2+^ Ions from the Resulting Ceramics

**Figure 4:**
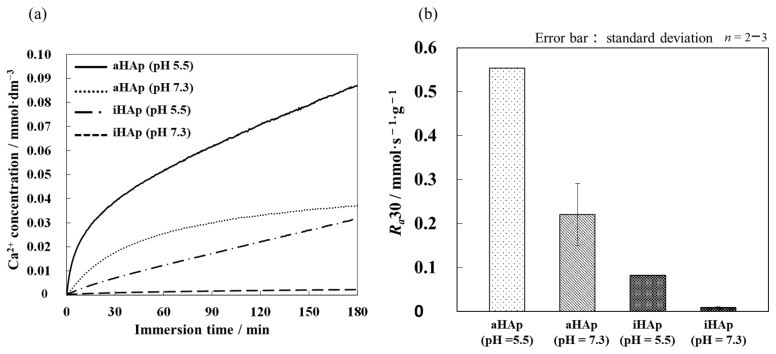
Dissolution tests of the resulting HAp ceramics using a Ca^2+^ ion-selective electrode under acidic and neutral conditions: (**a**) dissolution behavior of Ca^2+^ ions over 180 min when immersed in a solution and (**b**) the average dissolution rate of both ceramics under acidic and neutral conditions.

Figure 4a shows the change over time in the concentration of Ca^2+^ ions when iHAp and aHAp were immersed in acidic and neutral solutions, as defined in the Japanese Industrial Standards (JIS) T 0330-3 [28]. The results indicated that aHAp was more soluble in both environments compared with iHAp. Figure 4b shows the average dissolution rate per unit mass. Under acidic conditions, the rates in first 180 min dissolution rates of aHAp and iHAp reached 0.553 and 0.0824 mmol·s^−1^·g^−1^, respectively. In addition, under neutral conditions, the rate of aHAp and iHAp was 0.221 and 0.00928 mmol∙s^−1^∙g^−1^, respectively. Hence, the abovementioned results indicated that aHAp was about 6.7 times more soluble than iHAp when under acidic conditions. By contrast, aHAp was about 23 times more soluble than iHAp when under neutral conditions.

### 2.3. Determination of the Initial Cell-Adhesion Rate and Observation of Cell Morphology

**Figure 5:**
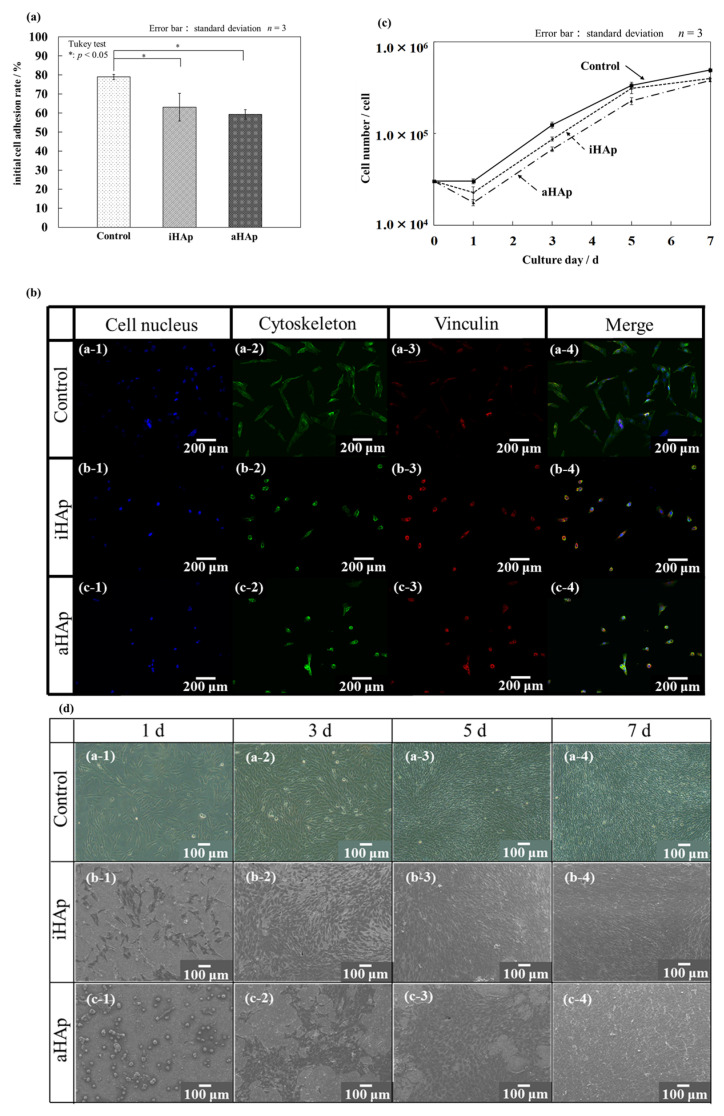
Initial cell-adhesion rates and growth behavior on fabricated ceramics. (**a**) Initial cell-adhesion rates. (**b**) Fluorescent immunostaining results for cells in the cell nucleus, cytoskeleton, and vinculin after 5 h of incubation ((**a-1**)–(**a-4**) control, (**b-1**)–(**b-4**) iHAp, and (**c-1**)–(**c-4**) aHAp). (**c**) Growth curves of cells cultured on ceramics. (**d**) Cell morphology after culturing on ceramics ((**a-1**)–(**a-4**) control, (**b-1**)–(**b-4**) iHAp, and (**c-1**)–(**c-4**) aHAp).

Figure 5a shows the initial cell-adhesion rate, which was measured by counting the number of cells 5 h after seeding. The results indicated that the initial cell-adhesion rates for the control group (polystyrene plate), iHAp, and aHAp reached 79.0%, 63.1%, and 59.3%, respectively. Thus, the initial cell-adhesion rate of aHAp was slightly lower than that of iHAp.

The cell morphology at the time of measuring the initial cell-adhesion rate was observed by fluorescent staining, and the results are shown in Figure 5b. The cells in the control group were already elongated after 5 h (Figure 5b(a-1)–(a-4)). On the contrary, the cells seeded on the ceramics had a spherical morphology (aggregation) after 5 h, and vinculin, which is a membrane protein that is part of the adhesive apparatus of cell adhesion, was barely observed. In addition, Figure 5c shows the growth curves of cells cultured on ceramics. The results on day 1 of culture tended to be higher for iHAp than for aHAp, which is similar to the initial adhesion rates.

Figure 5d shows the phase-contrast microscopic images of the control group and SEM images of cells cultured on individual ceramics. In the control group, elongated cells were observed from the first day of culture. On the contrary, the cells on aHAp shown in Figure 5d(c-1) had a spherical morphology (aggregation), which is similar to the fluorescent staining results of the initial cell-adhesion test, and proliferated cells were observed on all ceramics after day 5 of culture. Scattered forms of cell aggregation on aHAp were observed (Figure 5d(c-2),(c-3)). These results may be related to the low initial adherence (Figure 5a). Therefore, after a few days in culture, the cells in the control group and iHAp adhered and spread relatively early, proliferated, and formed sheets, whereas the cells in aHAp may adhere with an aggregated form, proliferate, and spread gradually.

### 2.4. Bone Differentiation of Osteoblasts Cultured on aHAp

**Figure 6:**
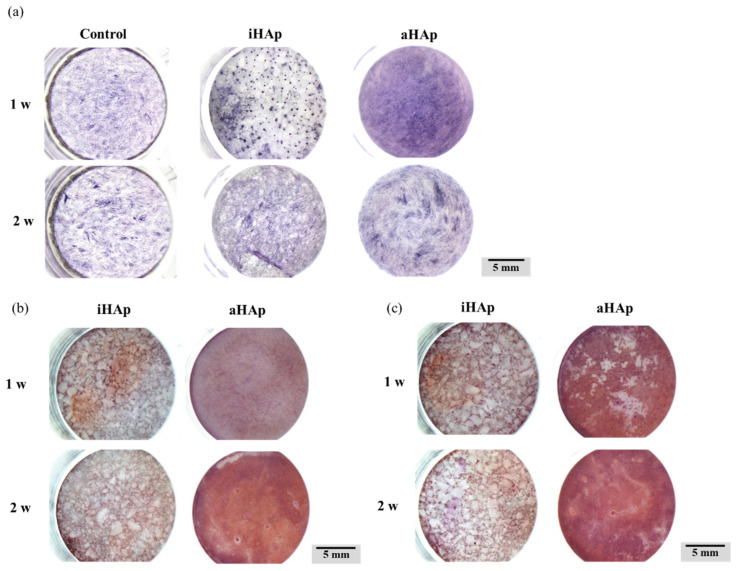
Histological evaluations using ALP and ARS staining for cultured cells on fabricated ceramics. (**a**) ALP staining for cultured specimens using a bone differentiation induction medium for 1–2 weeks after preculture. ARS staining (**b**) without and (**c**) with a bone differentiation induction medium for 1–2 weeks after preculture.

Figure 6a shows the results of alkaline phosphatase (ALP) staining for MG-63 cultured on each specimen. Darkly stained ALP-positive cells were observed in aHAp compared with other specimens. In addition, more darkly stained cells were observed in the group that used a bone differentiation induction medium for one week after preculture.

The results of alizarin red S (ARS) staining indicated an increase in calcification in aHAp, similar to the ALP staining results (Figure 6b,c). In particular, calcified areas were observed in aHAp with and without bone differentiation induction. Furthermore, the results indicated no differences in the aHAp group with or without bone differentiation induction.

**Figure 7:**
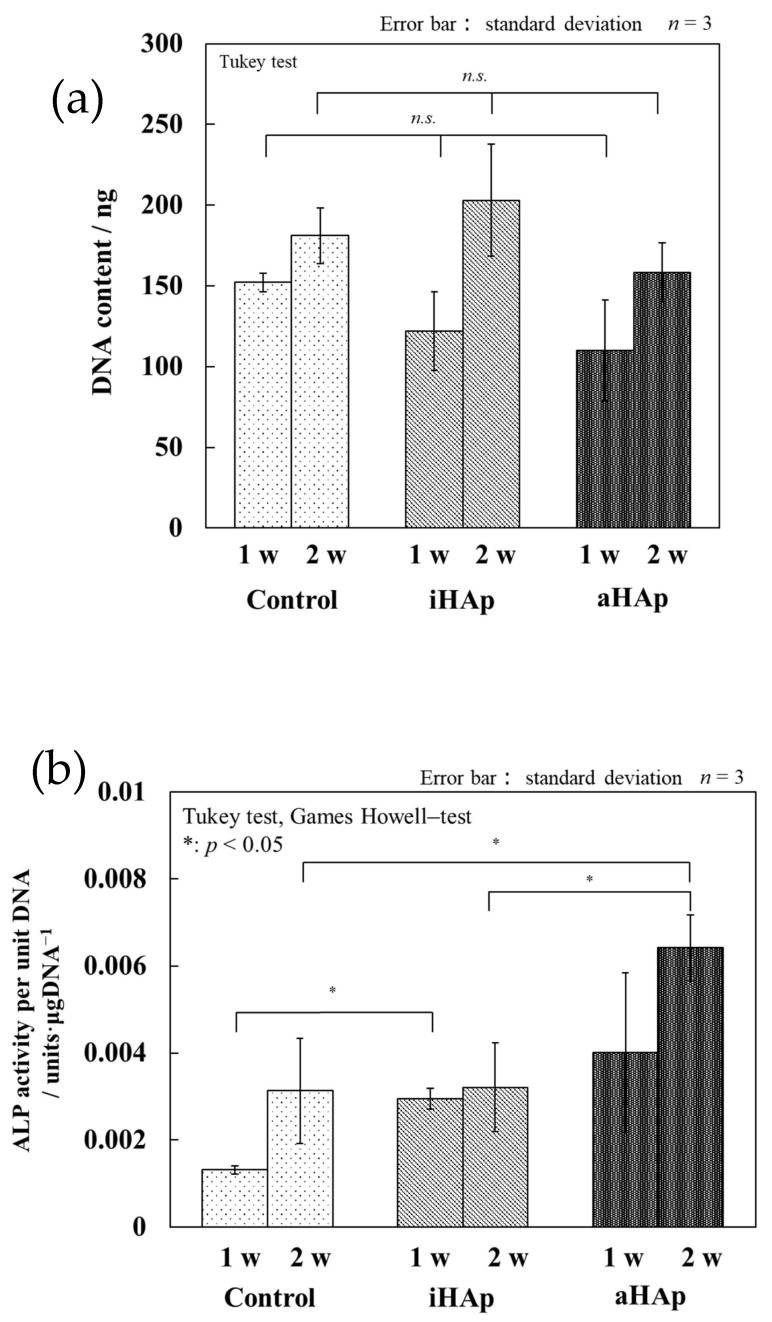
Quantitative evaluation of the amount of (**a**) DNA and (**b**) ALP activity in specimens after culturing (No significant difference is indicated by “*n.s.*”).

Figure 7a shows the DNA content of MG-63 cultured in an osteo-differentiation environment on each specimen. The amount of DNA tended to increase during culture. However, no significant differences were observed among the cultured specimens at each culture week. The ALP activity values of MG-63 cultured on each specimen are shown in Figure 7b. Significant differences were observed between the control group and iHAp at one week of incubation using a bone differentiation induction medium. By contrast, no significant differences were observed between iHAp and aHAp. After using a bone differentiation induction medium for two weeks, aHAp showed the highest value, with significant differences between aHAp and the control group, as well as between aHAp and iHAp.

### 2.5. Examination of Proteins Derived from Cells Cultured on aHAp by 2D Electrophoresis

**Figure 8:**
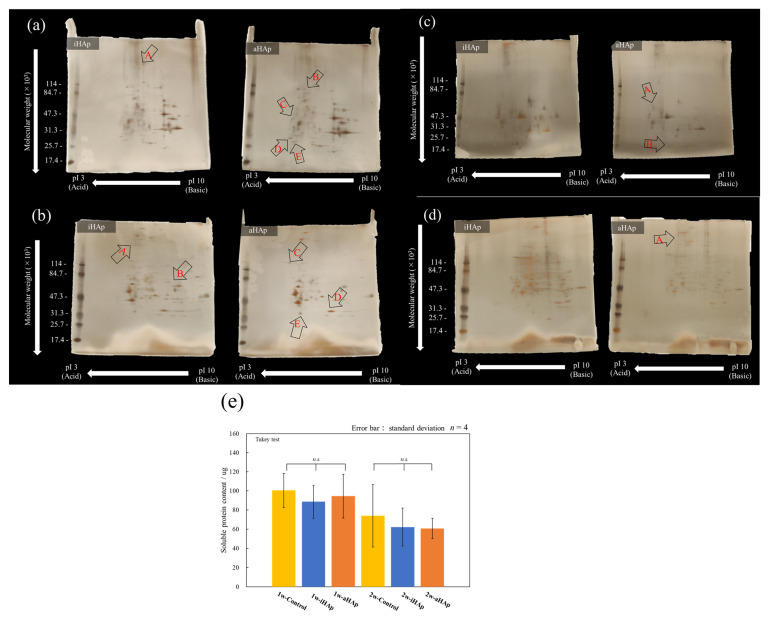
2D electrophoresis gel images of (**a**,**c**) soluble and (**b**,**d**) insoluble proteins obtained from cells cultured for 1 week (**a**,**b**) or 2 weeks (**c**,**d**) using a bone differentiation induction medium. (**e**) Quantitative evaluation of the soluble proteins obtained from cultured cells (No significant difference is indicated by “*n.s.*”).

Figure 8 shows the results of 2D electrophoresis of soluble and insoluble proteins on cells cultured for one and two weeks in an environment using a bone differentiation induction medium after preculture. Spots (A–E), which were expressed in only one of the samples, are indicated in the results.

The results of the one-week culture are shown in Figure 8a,b. The area indicated by “A” was observed only for iHAp. On the contrary, the proteins expressed at the sites shown in “B–E” were enriched in aHAp. In addition, the results for the insoluble proteins shown in Figure 8b indicated that “A” and “B” were more intensely expressed than aHAp, whereas the locations shown in “C–E” were more intensely expressed than iHAp.

Figure 8c,d show the 2D electrophoresis results of soluble and insoluble proteins on cells cultured for two weeks in an environment conducive to bone differentiation. As shown in Figure 8c, the areas shown in “A” and “B” were stained with aHAp. Similarly, as shown in Figure 8d, the area indicated by “A” was stained with aHAp. Figure 8e shows the results of measuring only soluble protein concentrations in the samples used for 2D electrophoresis. No significant difference was found in the concentration of soluble proteins collected among the samples.

## 3. Discussion

Apart from the typical HAp functional groups, AF and AG-AF had a CO_3_ group [29]. The CO_3_ group was derived from a homogeneous precipitation method of synthesis [25,26]. In addition, AG-AF had unsharp peaks of the AG-AF XRD pattern compared with AF because of the precipitation of HAp microcrystals. The peak intensity corresponding to (300) of the HAp crystal in iHAp was approximately 60% of the peak at (211), which is the strongest line, in accordance with the HAp peaks listed in ICDD-PDF No. 00-009-0432. Therefore, iHAp could be used as a control group in this study. As shown in Figure 2a, the peak corresponding to (300) of aHAp was higher than that of iHAp. Then, with regard to the fabricated ceramics (aHAp and iHAp), the crystalline orientation was visualized by EBSD. The EBSD results (Figure 3) indicated that aHAp was bluer, corresponding to (300) of the HAp crystal structure. Therefore, the fabricated aHAp had grains with *a*-planes exposed on the surface. In addition, iHAp showed scattered grains, indicating the *a*-plane of HAp, as well as *c*-planes and middle planes. These results indicate the isotropic structure of iHAp. Furthermore, the size of the crystal grains was larger in aHAp than in iHAp (Figure 3a). This difference in size may be due to the fact that aHAp was fabricated using the TGG method, which affects grain growth [30,31]. Image analysis of the EBSD measurement results shown in Figure 3b,c quantified that aHAp had more *a*-planes exposed than iHAp. aHAp also maintained a high relative density and exposed many *a*-planes. Therefore, it was suitable as a model ceramic for this study.

Under acidic and neutral conditions, the average dissolution rate per unit mass of aHAp was higher than iHAp. Therefore, solubility may be increased by exposing more *a*-planes in the general in vivo environment and in conditions that simulate the effect of osteoclasts. In general, the *a*-plane of HAp contains a large amount of calcium atoms. Thus, when *a*-planes are preferentially exposed, more Ca^2+^ ions are located at the surfaces. Since Ca has a high ionization tendency, it can be ionized more easily, which may increase the solubility of aHAp by exposing more *a*-planes. Furthermore, this phenomenon may be due to the microstructure of the AF used as a starting material; when AF is sintered, a void is observed in its crystals [32]. The surfaces of the voids could serve as additional dissolution sites.

The initial cell-adhesion rates shown in Figure 5a were higher for iHAp than for aHAp, and such a trend has been reported in our previous study [26]. The surface potential of aHAp was relatively higher than that of iHAp, and the contact angle of aHAp was higher than that of iHAp. Furthermore, the behavior of the adsorbed proteins differed between the two materials [18,27,33]. Bovine serum albumin (BSA), which is present in fetal bovine serum (FBS), serves as a typical example of an acidic protein. Notably, aHAp has shown a considerable capacity for the adsorption of acidic proteins [27]. Previous reports indicated that BSA can inhibit cell adhesion, which may contribute to the observed decrease in initial adhesion [34]. These differences in surface properties may affect the initial cell-adhesion rate. The results of cell proliferation were similar to the initial attachment rate, with iHAp having a higher number of cells than aHAp. The doubling times calculated on the cell numbers from days 1 to 5 were at 27.8, 25.4, and 26.1 h for the control group, iHAp, and aHAp, respectively. Wiecheć et al. reported a doubling time of 29.5 ± 3.7 h for MG-63 cells cultured on polystyrene plates [35]. Therefore, the cell-proliferation potential was almost the same between aHAp and iHAp. Furthermore, changes in cell numbers after day 5 of culture were small. The low number of cells in aHAp on day 1 of the cell-proliferation test was related to the low initial adhesion (Figure 5b). As shown in Figure 5d, the cells on aHAp were aggregated. Therefore, the cells in the control group and iHAp adhered and extended relatively early, proliferated, and formed sheets, whereas those in aHAp may adhere in a spherical form (aggregation), proliferate, and extend gradually.

With regard to osteogenic potential, darkly stained cells were observed on aHAp after culturing for one week based on the results of ALP staining (Figure 6a). Furthermore, quantitative evaluation indicated that the ALP activity of aHAp was significantly higher than that of iHAp (Figure 7b). Based on these results, the maximum ALP activity may occur for 1–2 weeks after using a bone differentiation induction medium.

**Figure 9:**
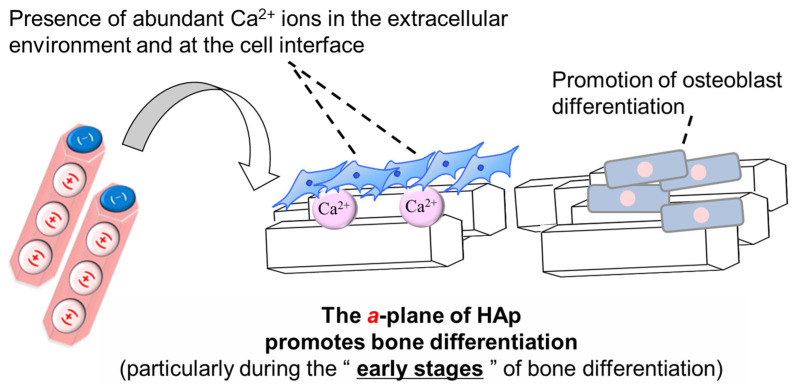
Mechanism by which aHAp promotes bone differentiation.

The results of ARS staining indicated that aHAp promoted osteoblast differentiation compared with iHAp. In particular, aHAp promoted bone differentiation compared with iHAp, even without a bone differentiation induction medium. Therefore, the *a*-plane of HAp promotes bone differentiation, especially in the early stages of bone differentiation. These results may be related to the Ca^2+^ ion concentrations shown in Figure 4. Maeno et al. reported that moderate Ca^2+^ ion concentrations promoted osteoblast differentiation [36]. According to Yanai et al., Ca^2+^ ions in the extracellular environment stimulated the mesenchymal stem cells of the nuclear factor of activated T cell signaling pathway, thereby influencing bone differentiation [37]. The results of their study were similar to those of the present study. In addition, the increased Ca^2+^ ion concentration at the interface between the aHAp surface and cells may induce bone differentiation (Figure 9). Yanagi et al. also reported the effect of bone regeneration using adipose-derived mesenchymal stem cells in monolayer and 3D spheroidal cultures. The results indicated that cells cultured in spheroid form had higher osteogenic potential than those cultured in a monolayer form [38]. Therefore, the ability of aHAp to promote cell aggregation may be a factor influencing the effect of aHAp on bone differentiation.

We also focused on the differences in the expression levels of proteins. The electrophoresis results indicated the differences in the expression levels of proteins between aHAp and iHAp (Figure 8). Nakanishi et al. reported that different cellular arrangements can affect molecular mechanisms, such as adhesive plaque activation [39]. Hayashi et al. also reported that not only the ionic concentration around the substrate but also the amount of protein adsorbed to the substrate can affect the osteogenic differentiation of mesenchymal stem cells [40]. Therefore, the difference in bone differentiation behavior is not only due to the ions found in the extracellular environment, but also due to the proteins secreted by cells caused by cell–cell interactions and proteins adsorbed to the substrate. Despite difficulty in identifying the expressed proteins using the method proposed in this study, different HAp orientations may affect the adsorption of proteins on the ceramics and the expression levels of proteins obtained from cultured cells because of the different amounts of eluted ions and different surface potentials of the ceramics [18,26,27]. Previous reports have shown that aHAp has a higher adsorption capacity for acidic proteins than iHAp [18,27,33]. In this study, no difference in the expression levels of soluble proteins was found, but the expression levels of acidic proteins tended to be higher in aHAp based on the results of 2D electrophoresis. Previous reports suggest that proteins involved in promoting bone formation include SFRP-3, an extracellular protein that affects Wnt signal transduction, and vitamin K epoxide reductase complex subunit 1 (VKORC1), which is involved in vitamin K metabolism [27]. Hence, this study focused on the relationship between the Ca^2+^ ion concentration derived from material properties and cell responses; however, the interface between cells and the adsorbed proteins is important. Such an interface may influence bone differentiation, and thus it will be the focus of future work [18,27].

## 4. Materials and Methods

### 4.1. Fabrication of aHAp

AF was prepared as a starting powder for aHAp, in accordance with a previous study [32]. After washing the synthesized AF, 1 mass% AF slurry was prepared, and AG-AF was synthesized in accordance with previous reports [26]. After 24 h, the AG-AF was washed three times with purified water, filtered by suction, washed with acetone to facilitate drying, and dried in an oven at 110 °C. Then, the AG-AF was milled and sieved twice through a mesh with an opening size of 500 μm to obtain the starting powder for aHAp. Based on previous findings, 30 mass% of low-crystallinity apatite gel was added to AF in order to expose more *a*-planes on the surface of aHAp and to increase a relative density of aHAp [26]. The crystal phase of the AG-AF was determined by XRD (MiniFlex, Rigaku Co., Tokyo, Japan). For comparison purposes, HAp-100 powder from Taihei Chemical Industry Co. was used. The functional groups were identified by using FTIR (IR Prestige-21, Shimadzu Corporation), with a spectral resolution of 4 cm^−1^ and a spectral range of 400–4000 cm^−1^. Measurements were performed using the KBr method by preparing measurement specimens. The morphology of the AF, AG-AF, and HAp-100 powders was observed using SEM (JSM-6390LA, JEOL Ltd., Tokyo, Japan). The AG-AF and HAp-100 powders (1.0 g) were filled in molding dies and uniaxially pressed using the Newton Press (NT-100H, NPA Systems, Saitama, Japan). AG-AF was compacted using a 17.5 mm-diameter molding machine at 200 MPa, whereas the HAp-100 powder was compacted in a 21.0 mm-diameter molding machine at 50 MPa.

The green compacts were sintered in a tube furnace (KTF433N, Koyo Thermo System, Nara, Japana), and the ceramics were fabricated. The sintering conditions were as follows: temperature increase rate of 5 °C·min^−1^, the green compact using AG-AF was sintered at 1300 °C, the green compact using HAp-100 powder was sintered at 1200 °C, and a holding time of 5 h in a steam atmosphere. The ceramics made of HAp-100 powder and AG-AF were referred to as iHAp and aHAp, respectively.

The material properties of the resulting ceramics were also characterized by using XRD, FTIR, and SEM. The XRD measurement for the fabricated ceramics was performed in the bulk state. The ceramics were polished on an automatic polishing machine (BUEHLER, Lake Bluff, IL, USA) using a water-resistant abrasive paper to prevent the effect of surface roughness on the cell response. The surface roughness of the polished ceramics was measured (S-3000, Mitutoyo, Kanagawa, Japan) and confirmed to be less than *R*_a_ = 0.1 mm. Then, the polished ceramics were ultrasonically cleaned with ethanol solution.

### 4.2. Measurement of the Degree of Orientation of aHAp

The crystal orientation of the resulting ceramics was evaluated using EBSD to obtain the orientation distribution map. In preparing the specimens, they were attached to glass slides with mounting wax and cut into 5 mm × 5 mm using a diamond cutter (BUEHLER, Lake Bluff, IL, USA). The specimens were polished on an automatic polisher using a #400 and #1000 water-resistant abrasive paper (Sankyo Rikagaku, Saitama, Japan). The polished surface of the specimen was sputtered using a cross-section polisher (IB-09020CP, JEOL, Tokyo, Japan). The sputtering conditions were as follows: Ar^+^ beam, 5.0 kV accelerating voltage, 3° beam angle of incidence (for surface observation), 2 h sputtering time for the surface, and 2.5 h sputtering time for the cross-section.

After polishing, the specimens underwent osmium deposition using an osmium coater (Neoc-ST, Maywa Forssys, Tokyo, Japan). EBSD was conducted using FE-SEM (JIB-4601, JEOL, Tokyo, Japan), EBSD (NordlysNano, Oxford, Abingdon-on-Thames, England), and EBSD software (AztecHKL, Oxford Instruments, Version: 3.1SP1) under the following conditions: 15.0 kV accelerating voltage, 70° sample tilt angle, and 18.0 mm distance between the sample and electron gun.

In quantifying the degree of orientation, image analysis of the EBSD data was performed. The EBSD images were color extracted using image analysis software (WinROOF, Mitani Trading, Version: 6.4.0) by classifying the crystal planes with exposed crystal grains of iHAp and aHAp crystals into the *a*-plane, *c*-plane, and the plane between the *a*-plane and *c*-plane.

### 4.3. Ca^2+^ Dissolution Behavior from aHAp

Dissolution tests (JIS T 0330-3) were performed on the fabricated ceramics using a Ca^2+^ ion-selective electrode under acidic and neutral conditions. In the acidic condition, an acetic acid–sodium acetate buffer solution (pH 5.50) was used, whereas in the neutral condition, a trishydroxymethylaminomethane hydrochloric acid buffer solution (pH 7.30) was used as the solvent. HORIBA 8203-10C was used as the calcium ion electrode, and HORIBA F-55 was used as the ion meter. The average dissolution rates, *R*_a_*t* (nmol·s^−1^) at *t* (min), during the measurement time were calculated as follows:

(1) $$R_{a}t=\frac{Ca\;ion\;concentration\;after\;t\;sec\;\left[mg\cdot {dm}^{-3}\right]\times liquid\;volume\;[{dm}^{3}]\times 10^{6}}{10\times molar\cdot mass\cdot of\cdot Ca\;\left[mg\cdot {mmol}^{-1}\right]\times t\;\left[min\right]\times 60\;[s\cdot {min}^{-1}]}$$

The calculation was performed under the following conditions: *t* = 30 min, liquid volume = 0.2 dm^3^, and molar mass of Ca = 40.08 mmg·mmol^−1^.

### 4.4. Assessment of Cellular Responsiveness to Hydroxyapatite Ceramics’ Preferred Orientation to the a-Plane

Human osteosarcoma-derived osteoblasts (MG-63) were cultured on aHAp and iHAp to examine the cell responsiveness of the ceramics. Polystyrene plates were used as a control. Sigma-Aldrich Minimum Essential Medium Eagle with 10 vol% FBS and 1 vol% nonessential amino acids (Sigma-Aldrich, St. Louis, MO, USA) was used as the medium. This medium was referred to as the “normal medium”. The “bone differentiation induction medium” was prepared by adding 10 mmol·dm^−3^ β-sodium glycerophosphate, 50 μg·cm^−3^ L-ascorbic acid, and 100 nmol·dm^−3^ dexamethasone to the normal medium. The cells were cultured at 37 °C with 5% CO_2_ atmosphere in an incubator. The initial cell-adhesion rates of the cells to the two types of ceramics were measured. Next, the specimens were placed in 24-well plates and seeded with 1.0 × 10^5^ cells using the normal medium. The cells on the specimens were collected and counted after 5 h. The initial cell-adhesion rates (%) were determined by dividing the number of cells attached to each specimen by the number of cells seeded (1.0 × 10^5^ cells) as 100%.

A cell-proliferation test was also performed by seeding cells on the two HAp ceramics. The fabricated ceramics were placed in 24-well plates and seeded with 3.0 × 10^4^ cells. After 1, 3, 5, and 7 days of culture, the cells were treated with 0.25 *w*/*v*% trypsin-EDTA solution and then removed from the specimen. Finally, the cells were counted.

### 4.5. Morphological Observation of Cells Cultured on HAp Ceramics

Cell morphology was observed using a fluorescent antibody technique in the initial cell-adhesion rate test. The specimens were washed with phosphate-buffered saline (PBS), and the cells were fixed in 4 mass% paraformaldehyde phosphate buffer solution (PFA, Wako Pure Chemical Industries, Osaka, Japan) at 4 °C. After washing, the cells were permeabilized using 0.25% Triton X-100/PBS solution and allowed to stand for 10 min. Afterward, the cells were washed with PBS and allowed to stand for 1 h in 3 *w*/*v*% BSA. Then, a primary antibody reaction was performed to stain vinculin. Anti-vinculin antibody [hVIN-1] ab11194 (isotype: mouse IgG1) was used as the primary antibody, and the process was performed overnight at 4 °C. Afterward, the specimens were incubated for 1 h with fluorescent reagents and a secondary antibody. At this time, the cell nuclei and cytoskeleton were stained with 4′,6-diamino-2-phenylindole and Alexa Fluor^®^ 488 phalloidin (Thermo Scientific, Waltham, MA, USA). Vinculin with Alexa Fluor^®^ 594Goat Anti-Mouse IgG1 (Thermo Scientific, Waltham, MA, USA) was used as the secondary antibody to stain vinculin. Furthermore, the specimens were observed using the Olympus BX51 epifluorescence system and FV300 confocal laser microscope (Tokyo, Japan).

After the cell-proliferation test, cell morphology was observed by SEM, and the morphology of cells cultured on the polystyrene plates (control) was observed using an Olympus (Tokyo, Japan) inverted microscope (CKX41). At 1, 3, 5, and 7 days after seeding, the cultured specimens were washed with PBS and fixed in PBS containing 10 vol% glutaraldehyde prepared using 25 wt% glutaraldehyde solution (Wako Pure Chemicals, Osaka, Japan) at 4 °C. The glutaraldehyde solution was discarded and washed with PBS. Then, the treated specimens were frozen in liquid nitrogen. After freeze drying, Pt was deposited and observed by using SEM.

### 4.6. Qualitative Evaluation of Bone Differentiation by ALP and ARS Staining

The bone differentiation levels of cells on the specimen were examined by ALP and ARS staining using a bone differentiation induction medium. After 5 days of preculture using a normal medium, the medium was switched to a bone differentiation induction medium, and the cells were further cultured for 1–2 weeks. Afterward, the specimens were washed with PBS. Then, the cells were fixed with PFA, and permeabilization was performed, as described in Section 4.5. For ALP staining, the cells were incubated with ALP substrate solution (Wako Pure Chemical Industries, Osaka, Japan) in an incubator at 37 °C for 30 min. Then, the cells were washed with sterile water and observed. ARS staining was also performed by adding 1 cm^3^ of ARS staining liquid (raw powder was made by Wako Pure Chemical Industries) and allowing it to stand for 5 min. Finally, the cells were washed again with sterile water, 95 vol% ethanol, and 100% ethanol and observed.

### 4.7. Quantitative Evaluation of DNA Content and ALP Activity

After culturing for 1–2 weeks using a bone differentiation induction medium, the collected cells were suspended in 20 mmol·dm^−3^ HEPES buffer. Subsequently, the cells were washed by centrifugation at 1000 rpm for 5 min. The supernatant fluid was discarded and suspended in 0.5 cm^3^ of HEPES buffer to prepare a cell suspension. In preparing a sample solution for DNA quantification, the cell suspension was homogenized by using an ultrasonic homogenizer (UD-200, TOMY SEIKO Co., Tokyo, Japan). In addition, 1 cm^3^ of HEPES buffer was added to 0.25 cm^3^ of the homogenized solution. Afterward, 0.75 cm^3^ of Hoechst 33,258 fluorescent reagent was added to 0.75 cm^3^ of the prepared sample solution, which was a solution diluted to suit the measurement, and 0.15 cm^3^ of this solution was placed in a quartz cuvette. Furthermore, the fluorescence intensity was measured at an excitation wavelength of 360 nm and a fluorescence wavelength of 460 nm using a spectrofluorometer (F-2700, Hitachi High-Tech Science Ltd., Tokyo, Japan). Standard reagents were prepared by diluting Salmon Sperm-derived DNA (Wako Pure Chemical Industries, Osaka, Japan) with HEPES buffer, as appropriate. The amount of DNA in each sample was determined from the relationship between the amount of DNA and the fluorescence intensity.

Then, ALP activity was measured using Laboassay™ ALP (Wako Pure Chemical Industries, Osaka, Japan) with the prepared cell suspension as the sample solution in accordance with the manufacturing protocol. Absorbance was measured at an excitation wavelength of 405 nm using a microplate reader (Multiskan FC, Thermo Scientific, Waltham, MA, USA).

### 4.8. Evaluation of Protein Expression in Cells Cultured on aHAp

Cells were seeded as described in Section 4.6 to examine protein expression. After culturing for 1–2 weeks in a bone differentiation induction medium, the cells were collected. Subsequently, the cell mass in suspension (as described in Section 4.6) was washed twice with a commercial buffer (EzApply 2D Kit, ATTO, Tokyo, Japan). After washing, the cells were centrifuged. The resulting cell fragments were suspended in 1.0 cm^3^ of Sample Preparation Solution 1 (EzApply 2D Kit, ATTO, Tokyo, Japan) and homogenized on ice using an ultrasonic homogenizer (UD-200, TOMY SEIKO CO., Tokyo, Japan). Following centrifugation, this solution was used as the soluble protein solution. The remaining cell sediments were suspended in 0.5 cm^3^ of Sample Preparation Solution 2 (EzApply 2D Kit, ATTO, Tokyo, Japan) and homogenized by sonication. The homogenized cell suspension was then centrifuged at 14,000 rpm and 4 °C for 20 min, and the central portion of the supernatant was collected as the insoluble protein solution. The protein concentration of the sample used for the soluble protein analysis was quantified using the Bradford method.

Two-dimensional electrophoresis was performed using isoelectric focusing (IEF) gels (E-D520L, ATTO) and acrylamide 2D gradient gels (gradient concentration 5–20%, A-M310, ATTO) following the O’Farrell method, with some modifications based on previous studies [18,27,41]. The first-dimension IEF (WSE-1510, ATTO Co., Tokyo, Japan) was conducted according to the manufacturer’s protocol, running at 300 V for 4 h. The second-dimension electrophoresis (WSE-1150, ATTO Co.) was also performed following the manufacturer’s protocol, with a current not exceeding 21 mA per gel and a voltage gradually increased to 400 V. After electrophoresis, the gels were stained using a silver staining kit (AE-1360, ATTO Co., Tokyo, Japan) according to the manufacturer’s instructions.

### 4.9. Statistical Analysis

Each numerical value was presented as mean ± SD. Statistical significance was calculated using statistical processing software R (Version: R 4.4.0) and RStudio (Version: 2024.09.1+394). Different testing methods were used depending on the variance and number of groups, and the methods used are described in each figure.

## 5. Conclusions

This study verified whether the preferred orientation of HAp influences osteogenesis using unique HAp ceramics we created. From EBSD results, the surface of aHAp was visualized, and we found that many of the *a*-planes of HAp were exposed. Furthermore, the ALP staining results showed that aHAp promoted the initial differentiation of osteoblasts, and the ARS staining results also showed that it accelerated osteoblast calcification. The quantitative results for ALP activity clarified that aHAp significantly enhanced osteoblast differentiation. In addition, cells cultured on aHAp produced specific proteins. Therefore, the *a*-plane of HAp promoted osteoblast differentiation. The anisotropy of HAp also makes it possible to control cell function, which may lead to the development of new biomaterials.

## Data Availability

The raw data supporting the conclusions of this article will be made available by the authors upon request.

## References

[1] de Vrese M., Pfeuffer M., Roos N., Scholz-Ahrens K., Schrezenmeir J. (2010). The health aspects of milk. Improving the Safety and Quality of Milk.

[2] Ielo I., Calabrese G., De Luca G., Conoci S. (2022). Recent Advances in Hydroxyapatite-Based Biocomposites for Bone Tissue Regeneration in Orthopedics. Int. J. Mol. Sci..

[3] Nakano T., Kaibara K., Tabata Y., Nagata N., Enomoto S., Marukawa E., Umakoshi Y. (2002). Unique alignment and texture of biological apatite crystallites in typical calcified tissues analyzed by microbeam X-ray diffractometer system. Bone.

[4] Yokota T., Miki T., Honda M., Ikeda-Fukazawa T., Ishii K., Matsumoto M., Aizawa M. (2018). Fabrication and biological evaluation of hydroxyapatite ceramics including bone minerals. J. Ceram. Soc. Jpn..

[5] Suzuki K., Fukasawa J., Miura M., Lim P.N., Honda M., Matsuura T., Aizawa M. (2021). Influence of Culture Period on Osteoblast Differentiation of Tissue-Engineered Bone Constructed by Apatite-Fiber Scaffolds Using Radial-Flow Bioreactor. Int. J. Mol. Sci..

[6] Suzuki K., Honda M., Matsuura T., Aizawa M. (2022). Living reactions of tissue-engineered bone derived from apatite-fiber scaffold in rat subcutaneous tissues. J. Ceram. Soc. Jpn..

[7] Sila-Asna M., Bunyaratvej A., Maeda S., Kitaguchi H., Bunyaratavej N. (2007). Osteoblast differentiation and bone formation gene expression in strontium-inducing bone marrow mesenchymal stem cell. Kobe J. Med. Sci..

[8] Casarrubios L., Gomez-Cerezo N., Sanchez-Salcedo S., Feito M.J., Serrano M.C., Saiz-Pardo M., Ortega L., de Pablo D., Diaz-Guemes I., Fernandez-Tome B. (2020). Silicon substituted hydroxyapatite/VEGF scaffolds stimulate bone regeneration in osteoporotic sheep. Acta Biomater..

[9] Ishikawa K., Hayashi K. (2021). Carbonate apatite artificial bone. Sci. Technol. Adv. Mater..

[10] Ressler A., Žužić A., Ivanišević I., Kamboj N., Ivanković H. (2021). Ionic substituted hydroxyapatite for bone regeneration applications: A review. Open Ceram..

[11] Jarrar H., Çetin Altındal D., Gümüşderelioğlu M. (2021). Scaffold-based osteogenic dual delivery system with melatonin and BMP-2 releasing PLGA microparticles. Int. J. Pharm..

[12] Sheikh Z., Javaid M.A., Hamdan N., Hashmi R. (2015). Bone Regeneration Using Bone Morphogenetic Proteins and Various Biomaterial Carriers. Materials.

[13] Hustedt J.W., Blizzard D.J. (2014). The controversy surrounding bone morphogenetic proteins in the spine: A review of current research. Yale J. Biol. Med..

[14] Zhu L., Liu Y., Wang A., Zhu Z., Li Y., Zhu C., Che Z., Liu T., Liu H., Huang L. (2022). Application of BMP in Bone Tissue Engineering. Front. Bioeng. Biotechnol..

[15] Aizawa M., Howell F.S., Itatani K., Yokogawa Y., Nishizawa K., Toriyama M., Kameyama T. (2000). Fabrication of Porous Ceramics with Well-Controlled Open Pores by Sintering of Fibrous Hydroxyapatite Particles. J. Ceram. Soc. Jpn..

[16] Aizawa M., Matsuura T., Zhuang Z. (2013). Syntheses of Single-Crystal Apatite Particles with Preferred Orientation to the *a*- and *c*-Axes as Models of Hard Tissue and Their Applications. Biol. Pharm. Bull..

[17] Zhuang Z., Yoshimura H., Aizawa M. (2013). Synthesis and ultrastructure of plate-like apatite single crystals as a model for tooth enamel. Mater. Sci. Eng. C Mater. Biol. Appl..

[18] Onuma E., Ito H., Sasaki M., Kanzawa N., Kito K., Aizawa M. (2023). Proteomics of serum proteins adsorbed onto hydroxyapatite single-crystal particles with an anisotropic structure. Materialia.

[19] Ioku K. (1999). Hydrothermal Preparation of Advanced Phosphate Materials. Inorg. Mater..

[20] Zhang H., Darvell B.W. (2011). Morphology and structural characteristics of hydroxyapatite whiskers: Effect of the initial Ca concentration, Ca/P ratio and pH. Acta Biomater..

[21] Ban S., Hasegawa J. (2002). Morphological regulation and crystal growth of hydrothermal-electrochemically deposited apatite. Biomaterials.

[22] Inagaki M., Kameyama T. (2007). Phase transformation of plasma-sprayed hydroxyapatite coating with preferred crystalline orientation. Biomaterials.

[23] Ban S., Maruno S. (1998). Morphology and microstructure of electrochemically deposited calcium phosphates in a modified simulated body fluid. Biomaterials.

[24] Sakai T., Ueshima M., Morita S., Nakamura S., Yamashita K. (2011). Biological Reaction to Electrically Polarized Hydroxyapatite. MRS Online Proc. Libr..

[25] Aizawa M., Kinoshita M., Yamada K., Itatani K., Kishioka A. (1998). Effects of Additives on Synthesis and Morphology of Carbonate–Containing Hydroxyapatite Prepared by Homogeneous Precipitation Method. Inorg. Mater..

[26] Zhuang Z., Fujimi T.J., Nakamura M., Konishi T., Yoshimura H., Aizawa M. (2013). Development of *a*,*b*-plane-oriented hydroxyapatite ceramics as models for living bones and their cell adhesion behavior. Acta Biomater..

[27] Onuma E., Honda T., Yoshimura H., Nishihara T., Ogura A., Kanzawa N., Aizawa M. (2023). Identification of Proteins Adsorbed on Hydroxyapatite Ceramics with a Preferred Orientation to *a*-Plane. Crystals.

[28] Ito A., Sogo Y., Yamazaki A., Aizawa M., Osaka A., Hayakawa S., Kikuchi M., Yamashita K., Tanaka Y., Tadokoro M. (2015). Interlaboratory studies on in vitro test methods for estimating in vivo resorption of calcium phosphate ceramics. Acta Biomater.

[29] Anjaneyulu U., Priyadarshini B., Stango A.X., Chellappa M., Manivasagam G., Vijayalakshmi D. (2017). Preparation and characterisation of sol–gel-derived hydroxyapatite nanoparticles and its coatings on medical grade Ti-6Al-4V alloy for biomedical applications. Mater. Technol..

[30] Messing G.L., Trolier-McKinstry S., Sabolsky E.M., Duran C., Kwon S., Brahmaroutu B., Park P., Yilmaz H., Rehrig P.W., Eitel K.B. (2004). Templated Grain Growth of Textured Piezoelectric Ceramics. Crit. Rev. Solid State Mater. Sci..

[31] Akça E., Duran C., Kowalski B., Sehirlioglu A. (2021). Templated grain growth of Bi(Zn0.5Zr0.5)O3modified BiScO3−PbTiO3piezoelectric ceramics for high temperature applications. J. Asian Ceram. Soc..

[32] Aizawa M., Porter A.E., Best S.M., Bonfield W. (2005). Ultrastructural observation of single-crystal apatite fibres. Biomaterials.

[33] Zhuang Z., Aizawa M. (2013). Protein adsorption on single-crystal hydroxyapatite particles with preferred orientation to *a*(*b*)- and c-axes. J. Mater. Sci. Mater. Med..

[34] Kan M., Minamoto Y., Sunami S., Yamam I., Umeda M. (1982). The Effects on Cell Adhesion of Fibronectin and Gelatin in a Serum-Free, Bovine Serum Albumin Medium. Cell Struct. Funct..

[35] Wiecheć A., Stodolak-Zych E., Frączek-Szczypta A., Błażewicz M., Kwiatek W.M. (2012). The Study of Human Osteoblast-Like MG 63 Cells Proliferation on Resorbable Polymer-Based Nanocomposites Modified with Ceramic and Carbon Nanoparticles. ACTA Phys. Pol. A.

[36] Maeno S., Niki Y., Matsumoto H., Morioka H., Yatabe T., Funayama A., Toyama Y., Taguchi T., Tanaka J. (2005). The effect of calcium ion concentration on osteoblast viability, proliferation and differentiation in monolayer and 3D culture. Biomaterials.

[37] Yanai R., Tetsuo F., Ito S., Itsumi M., Yoshizumi J., Maki T., Mori Y., Kubota Y., Kajioka S. (2019). Extracellular calcium stimulates osteogenic differentiation of human adipose-derived stem cells by enhancing bone morphogenetic protein-2 expression. Cell Calcium.

[38] Yanagi T., Kajiya H., Fujisaki S., Maeshiba M., Yanagi S.A., Yamamoto M.N., Kakura K., Kido H., Ohno J. (2021). Three-dimensional spheroids of dedifferentiated fat cells enhance bone regeneration. Regen. Ther..

[39] Nakanishi Y., Matsugaki A., Kawahara K., Ninomiya T., Sawada H., Nakano T. (2019). Unique arrangement of bone matrix orthogonal to osteoblast alignment controlled by Tspan11-mediated focal adhesion assembly. Biomaterials.

[40] Hayashi K., Kishida R., Tsuchiya A., Ishikawa K. (2019). Honeycomb blocks composed of carbonate apatite, beta-tricalcium phosphate, and hydroxyapatite for bone regeneration: Effects of composition on biological responses. Mater. Today Bio.

[41] O’Farrell P.Z., Goodman H.M., O’Farrell P.H. (1977). High resolution two-dimensional electrophoresis of basic as well as acidic proteins. Cell.

